# Effects of microgravity on neural crest stem cells

**DOI:** 10.3389/fnins.2024.1379076

**Published:** 2024-03-27

**Authors:** Yilin Han, Povilas Barasa, Lukas Zeger, Sara B. Salomonsson, Federica Zanotti, Marcel Egli, Barbara Zavan, Martina Trentini, Gunnar Florin, Alf Vaerneus, Håkan Aldskogius, Robert Fredriksson, Elena N. Kozlova

**Affiliations:** ^1^Department of Immunology, Genetics and Pathology, Uppsala University, Uppsala, Sweden; ^2^Institute of Biochemistry, Vilnius University, Vilnius, Lithuania; ^3^Department of Pharmaceutical Bioscience, Uppsala University, Uppsala, Sweden; ^4^Department of Translational Medicine, University of Ferrara, Ferrara, Italy; ^5^Space Biology Group, School of Engineering and Architecture, Institute of Medical Engineering, Lucerne University of Applied Sciences and Arts, Hergiswil, Switzerland; ^6^National Center for Biomedical Research in Space, Innovation Cluster Space and Aviation, University of Zurich, Zurich, Switzerland; ^7^Swedish Space Corporation, Solna, Sweden

**Keywords:** microgravity, proliferation, delayed effect, neural stem cell, gene expression, exosomes, microRNA

## Abstract

Exposure to microgravity (μg) results in a range of systemic changes in the organism, but may also have beneficial cellular effects. In a previous study we detected increased proliferation capacity and upregulation of genes related to proliferation and survival in boundary cap neural crest stem cells (BC) after MASER14 sounding rocket flight compared to ground-based controls. However, whether these changes were due to μg or hypergravity was not clarified. In the current MASER15 experiment BCs were exposed simultaneously to μg and 1 g conditions provided by an onboard centrifuge. BCs exposed to μg displayed a markedly increased proliferation capacity compared to 1 g on board controls, and genetic analysis of BCs harvested 5 h after flight revealed an upregulation, specifically in μg-exposed BCs, of Zfp462 transcription factor, a key regulator of cell pluripotency and neuronal fate. This was associated with alterations in exosome microRNA content between μg and 1 g exposed MASER15 specimens. Since the specimens from MASER14 were obtained for analysis with 1 week’s delay, we examined whether gene expression and exosome content were different compared to the current MASER15 experiments, in which specimens were harvested 5 h after flight. The overall pattern of gene expression was different and Zfp462 expression was down-regulated in MASER14 BC μg compared to directly harvested specimens (MASER15). MicroRNA exosome content was markedly altered in medium harvested with delay compared to directly collected samples. In conclusion, our analysis indicates that even short exposure to μg alters gene expression, leading to increased BC capacity for proliferation and survival, lasting for a long time after μg exposure. With delayed harvest of specimens, a situation which may occur due to special post-flight circumstances, the exosome microRNA content is modified compared to fast specimen harvest, and the direct effects from μg exposure may be partially attenuated, whereas other effects can last for a long time after return to ground conditions.

## 1 Introduction

Exposure to microgravity (μg) results in a range of systemic changes in the organism, reflecting the physiological stress and adaptation associated with this extreme physical environment (Corydon et al., 2023). Space flight conditions have also been shown to alter brain structure and function with more severe effects after long-term space missions (Roy-O'Reilly et al., 2021; Shirah et al., 2022). Microgravity induces stress-related changes in cellular structure and gene expression (Corydon et al., 2023). However, μg also has some beneficial cellular effects and was shown to promote neuronal differentiation of mesenchymal stem cells (Chen et al., 2011), adipose stem cells (Zarrinpour et al., 2017; Graziano et al., 2018) and to promote cardiomyocyte development (Camberos et al., 2019).

Boundary cap neural crest stem cells (BCs) are a transient group of cells located at spinal root exit and entry points during embryonic development and can differentiate into neurons and glia (Hjerling-Leffler et al., 2005; Aldskogius et al., 2009; Trolle et al., 2014; Radomska and Topilko, 2017). In addition to their broad differentiation potential, BCs display a remarkable ability to promote survival and support the function of other cells (Olerud et al., 2009; Grouwels et al., 2012; Ngamjariyawat et al., 2013; Aggarwal et al., 2017). We previously showed that BCs analyzed 1 week after exposure to μg condition on the MASER14 sounding rocket flight markedly increased their post-flight proliferation capacity compared to ground controls, and showed activation of genes associated with survival and differentiation (Han et al., 2021). However, whether these changes were induced by exposure to μg or to hypergravity remain to be clarified. Furthermore, since space flown BCs were harvested after 1 week’s delay, the observed effects could be indirect, i.e., mediated through factors released by BCs to the medium as a result of the space flight conditions.

In the current experiment with MASER15 sounding rocket we asked (i) whether hypergravity or μg induces increased proliferation and altered gene expression in BCs; (ii) whether these effects are detectable also in BCs analyzed directly after the flight; and (iii) whether exosome content in the medium differs after direct harvest (MASER15 experiment) compared to delayed harvest (MASER14 experiment). To distinguish between the influence of μg and hypergravity, BCs were placed in two separate sections in the sounding rocket: one group was exposed to μg, whereas another group was placed in an onboard centrifuge that provided 1 g control condition.

The experiments revealed that exposure specifically to μg induces the increased BC proliferation capacity, which can be detected in specimens harvested shortly after flight and are associated with specific alterations in gene expression. Furthermore, distinct differences were identified in exosome microRNA content between MASER14 (1 week delayed harvest) and MASER15 (5 h delayed harvest).

## 2 Methods

### 2.1 Preparation and culture of boundary cap neural crest stem cells

The Regional Ethics Committee for Research on Animals approved all animal procedures. BCs were prepared from transgenic mice harboring red fluorescent protein (RFP) under the universal actin promoter as previously described and cultured under the same conditions (Aldskogius et al., 2009). Briefly, the spinal cord was exposed and dorsal root ganglia, including their attachment with the spinal cord, gently separated and mechano-enzymatically dissociated using collagenase/dispase (1 mg/mL) and DNase (0.5 mg/mL) for 30 min at room temperature. Cells were plated at 0.5–1 × 10^5^ cells/cm^2^ in N_2_ medium containing B27 (Gibco) as well as EGF and bFGF (R&D Systems; 20 ng/mL, respectively). After 12 h of culture, cells that had not adhered were removed together with half of the medium, and a fresh medium was added. The medium was changed every second day, and neurospheres could be observed after about 2 weeks of culture.

### 2.2 Assembly of specimens for insertion to the space module

The final preparations before launch of MASER15 were performed in the Esrange Space Center bio-laboratory.^1^ SIOUX Technologies^2^ provided the special hardware for storing the cells during space flight. For the assembly, a special metal tripod-table was used. All cellular materials were prepared as triplicates at around 0.3 M cells/ml density, placed to membranes within the hard-plastic building blocks. The membranes were sealed airtight with respective membrane lids and remaining bubbles were aspirated by injecting needles (G25, G27) through the membrane lids. Cassettes were installed in the “Late-access unit” (LAU), which functioned as an airtight, pressure-, temperature-controlled incubator aboard the rocket. The center part of the LAU was built as a centrifuge to mimic regular gravitational acceleration (1G), while the outer parts of the LAU were exposed to microgravity (weightlessness) during the flight. The ground control group was cultured in ambient temperature condition on the bench at Esrange throughout the flight until all materials returned to the lab.

**Figure 1:**
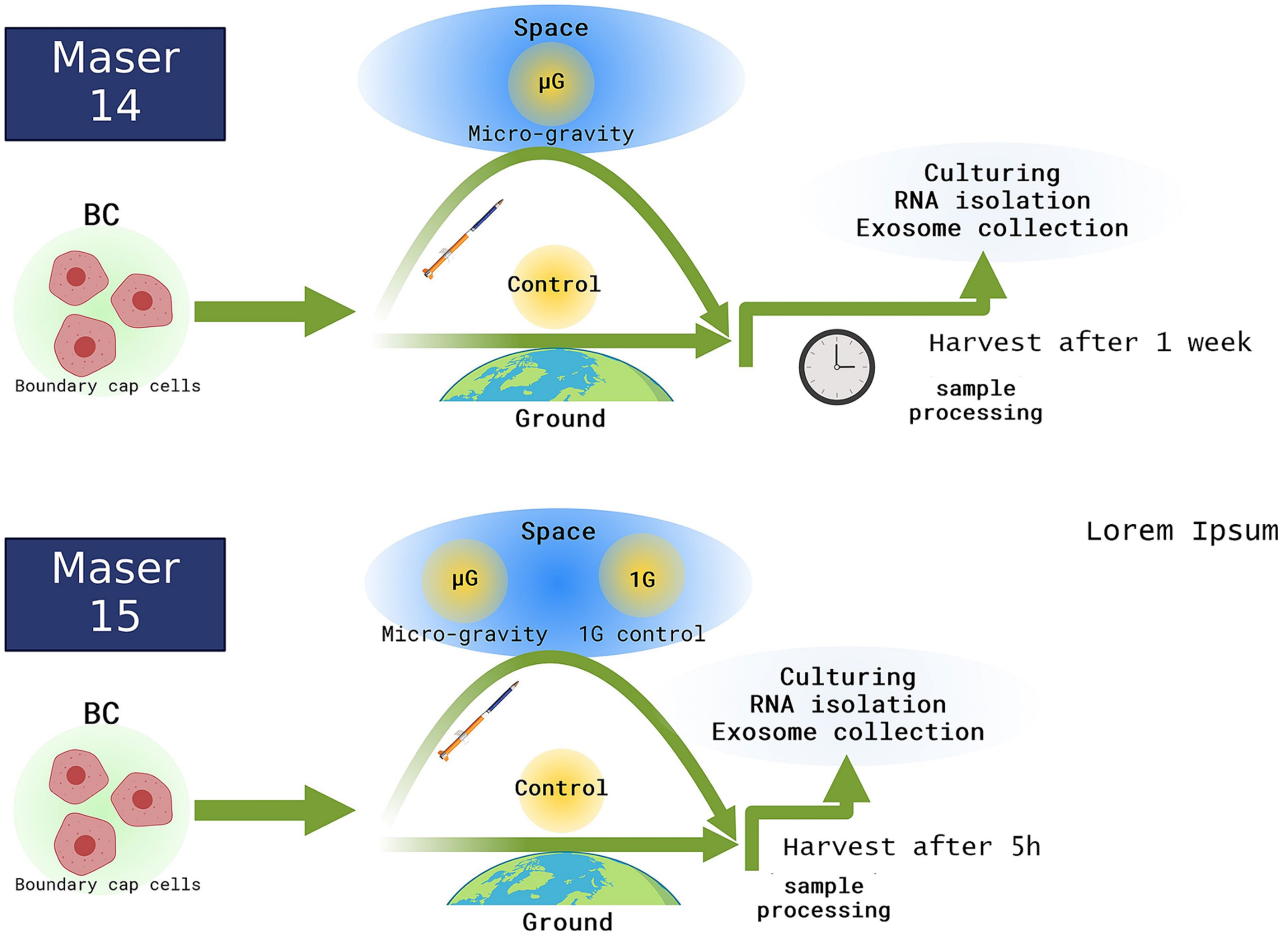
The overview and time line of the experiments in Space. BCs as floating spheres were prepared in the laboratory and placed to rocket 3 h before launch. In Maser 15 experiment just before entering space microgravity the centrifuge was started, maintaining 1G throughout flight whereas the rest of the cells were subjected to microgravity condition. After landing the cells were delivered to the laboratory and after Maser 15 cells were processed directly for analysis, whereas after Maser 14 the cells were retrieved for analysis 1 week after landing.

All samples for further analysis were divided into three groups; one part was placed in a centrifuge installed in the experimental module onboard to keep 1 g condition, another part was subjected to μg, and the third part remained on the ground as a control group (Figure 1, overview of the experiment).

Microgravity conditions with sounding rocket were achieved between 75 s (at 100 km altitude) and 438.1 s (at 110 km altitude) after lift-off, providing 363 0.4 s of microgravity (source: SSC document “S1X-3 M15 post flight report,” ref.: S1XM-384989335-2407, 24 November 2021).

Microgravity conditions are identified by measuring residual accelerations in the vehicle’s Service Module. For this, a set of calibrated and flight-qualified 3-axis accelerometers (Honeywell Q-Flex^®^ QA-1400 series) were used. Measurement range in fine mode were minus 60.0 mG to 60.0 mG with 4 μG resolution. Sampling frequency was 2,500 Hz (source: SSC document “MASM-2B design report,” ref.: SCIPROJ-1835638381-8199, 20 December 2022).

### 2.3 Post-flight culture and morphological assessment

Part of the cells subjected to μg or 1 g condition on MASER15 was retrieved from the membranes 5 h after landing for RNA extraction, whereas another group of cells received fresh medium and was placed into 24 -well plates for extension. The cells exposed to μg required every day split due to their extreme rate of proliferation, whereas cells in 1 g group and ground control cells were split every second day according to the standard protocol. This situation resembled the postflight conditions from the previous MASER14 BC experiment (Han et al., 2021) and prompted a comparison of the rate of BC proliferation between these two flights. For the comparison of BC proliferation rate, the neurospheres from Maser 14, Maser 15 and non-flight groups were split to single cells, stained with trypan blue, counted in Burker chamber and seeded to low-affinity 6 well-dishes with the equal concentrations. After 3 days the all cells were collected, split to the single cells and counted in the Burker chamber.

### 2.4 Transcriptomics analysis of BCs exposed to space flight

#### 2.4.1 AmpliSeq sequencing

For transcriptomic analysis, the medium was replaced immediately after cells were accessible for analysis (5 h after landing) with RNAlater^®^ (#R0901, Sigma-Aldrich), and samples were transported at room temperature and subsequently frozen at −80°C until processed for total RNA extraction using the Aurum™ Total RNA Mini Kit (#7326820, Bio-Rad) according to manufacturer’s instruction. Concentration was determined using NanoDrop^™^ 1000 (Thermo Fisher). Before sequencing, RNA was quality-controlled using a 2100 Bioanalyzer (Agilent). For sequencing, 10 ng of RNA from each sample was used. Sequencing was performed using an Ion S5^™^ XL system (Thermo Fisher). The data was processed through the ampliSeqRNA plugin in the Torrent Suite Software.

#### 2.4.2 Differential gene expression analysis

Analysis of gene expression data was performed using R 4.3.1 (Team, 2023) with the DESeq2 1.40.2 (Love et al., 2014) package. Differential expression analysis was performed on raw read counts from AmpliSeq, normalized with the median-of-ratios method in DESeq2. Differentially expressed genes (DEGs) with an absolute log2 fold change (log2FC) above 2.0 and false discovery rate (FDR) adjusted *p*-value < 0.001 were used for further analysis. The MASER15 cells exposed to μg were compared to ground control and 1 g control. For MASER14, cells exposed to space flight were compared to ground control.

Pathway enrichment analysis was performed for differentially expressed genes in MASER 15 and MASER 14 compared to the respective ground control group using the REACTOME database (Fabregat et al., 2017). Up- and downregulated genes were assessed separately. The top 100 pathways for each analysis were considered. Because similar sets of genes generated hits on multiple related pathways in REACTOME, the genes triggering these hits were manually combined in four non-redundant genes of interest lists (Tables 1–4).

**Table 1:** Genes of interest from MASER15, upregulated in cells exposed to space flight μg.

Gene symbol	Gene description	Assigned function
Antxr2	ANTXR Cell Adhesion Molecule 2	Cell adhesion
Camkk2	Calcium/Calmodulin Dependent Protein Kinase Kinase 2	Neuronal signaling
Ccnd1	Cyclin D1	Cell division
Chst7	Carbohydrate Sulfotransferase 7	Proteoglycan
Dph2	Diphthamide Biosynthesis 2	Cell cycle
Il20rb	Interleukin 20 Receptor Subunit Beta	Immune system
Kcng1	Potassium Voltage-Gated Channel Modifier Subfamily G Member 1	Neuronal signaling
Map3k11	Mitogen-Activated Protein Kinase Kinase Kinase 11	Cell division
Myc	MYC Proto-Oncogene, BHLH Transcription Factor	Cell division
Ptger4	Prostaglandin E Receptor 4	Immune system
Slc35b3	Solute Carrier Family 35 Member B3	Cell division
Snx9	Sorting Nexin 9	Vesicle biogenesis
Srxn1	Sulfiredoxin 1	Stress response
Tor1aip2	Torsin 1A Interacting Protein 2	Stress response
Tyw3	TRNA-YW Synthesizing Protein 3 Homolog	Cell division
Uroc1	Urocanate Hydratase 1	Histidine metabolism

**Table 2:** Genes of interest from MASER15, down-regulated in cells exposed to space flight μg.

Gene symbol	Gene description	Assigned function
Aldoa	Aldolase, Fructose-Bisphosphate A	Metabolism
Bbc3	BCL2 Binding Component 3	Apoptosis
Bmt2	Base Methyltransferase Of 25S RRNA 2 Homolog	Metabolism
Cbx4	Chromobox 4	Cell division
Cbx8	Chromobox 8	Cell division
Ccng2	Cyclin G2	Cell division
Cdkn1b	Cyclin Dependent Kinase Inhibitor 1B	Cell division
Chuk	Component Of Inhibitor Of Nuclear Factor Kappa B Kinase Complex	Immune system
Copb1	COPI Coat Complex Subunit Beta 1	Vesicle biogenesis
Crebrf	CREB3 Regulatory Factor	Cell division
Ddit4	DNA Damage Inducible Transcript 4	Hypoxia
Egln3	Egl-9 Family Hypoxia Inducible Factor 3	Hypoxia
Ero1l	Endoplasmic Reticulum Oxidoreductase 1 Alpha	Immune system
Gadd45b	Growth Arrest And DNA Damage Inducible Beta	Epigenetics
Gbe1	1,4-Alpha-Glucan Branching Enzyme 1	Proteoglycan synthesis
Gys1	Glycogen Synthase 1	Metabolism
Herpud1	Homocysteine Inducible ER Protein With Ubiquitin Like Domain 1	Apoptosis
Higd1a	HIG1 Hypoxia Inducible Domain Family Member 1A	Hypoxia
Hk2	Hexokinase 2	Metabolism
Hoxb2	Homeobox B2	Cell division
Mmaa	Metabolism Of Cobalamin Associated A	Metabolism
Nampt	Nicotinamide Phosphoribosyltransferase	Metabolism
Nfkbia	NFKB Inhibitor Alpha	Immune system
P4ha1	Prolyl 4-Hydroxylase Subunit Alpha 1	Collagen synthesis
Pfkl	Phosphofructokinase, Liver Type	Metabolism
Pgm2	Phosphoglucomutase 2	Metabolism
Plod2	Procollagen-Lysine,2-Oxoglutarate 5-Dioxygenase 2	Collagen synthesis
Pole3	DNA Polymerase Epsilon 3, Accessory Subunit	Collagen synthesis
Ppp1r3c	Protein Phosphatase 1 Regulatory Subunit 3C	Glycogen synthesis
Sap30	Sin3A Associated Protein 30	Epigenetics
Slc2a1	Solute Carrier Family 2 Member 1	Metabolism
Trib3	Tribbles Pseudokinase 3	Apoptosis
Ypel5	Yippee Like 5	Cell division

**Table 3:** Genes of interest from MASER14, upregulated in cells exposed to space flight.

Gene symbol	Gene description	Assigned function
A2m	Alpha-2-Macroglobulin	Immune system
Abca1	ATP Binding Cassette Subfamily A Member 1	Immune system
Adm2	Adrenomedullin 2	Other
Ank3	Ankyrin 3	Cell–Cell contact
Anxa2	Annexin A2	Neuronal signaling
Apobr	Apolipoprotein B Receptor	Metabolism
Appl2	Adaptor Protein, Phosphotyrosine Interacting With PH Domain And Leucine Zipper 2	Neuronal signaling
Asic4	Acid Sensing Ion Channel Subunit Family Member 4	Neuronal signaling
Atp11b	ATPase Phospholipid Transporting 11B (Putative)	Ion transport
Atp1a4	ATPase Na+/K+ Transporting Subunit Alpha 4	Ion transport
Atp8a2	ATPase Phospholipid Transporting 8A2	Ion transport
Atxn3	Ataxin 3	Cell division
C3	Complement C3	Immune system
C4b	Complement C4B (Chido Blood Group)	Development of nervous system
Camp	Cathelicidin Antimicrobial Peptide	Immune system
Cd68	CD68 Molecule	Immune system
Chrm2	Cholinergic Receptor Muscarinic 2	Neuronal signaling
Cldn10	Claudin 10	Cell–Cell contact
Cldn4	Claudin 4	Cell–Cell contact
Cldn7	Claudin 7	Cell–Cell contact
Clu	Clusterin	Metabolism
Cox6a2	Cytochrome C Oxidase Subunit 6A2	Mitochondria
Cp	Ceruloplasmin	Ion transport
Cpz	Carboxypeptidase Z	Metabolism
Crebrf	CREB3 Regulatory Factor	Cell division
Crispld2	Cysteine Rich Secretory Protein LCCL Domain Containing 2	Immune system
Ctsh	Cathepsin H	Other
Cyp26b1	Cytochrome P450 Family 26 Subfamily B Member 1	Other
Cyp7b1	Cytochrome P450 Family 7 Subfamily B Member 1	Other
Cystm1	Cysteine Rich Transmembrane Module Containing 1	Other
Dapk2	Death Associated Protein Kinase 2	Apoptosis
Dhrs3	Dehydrogenase/Reductase 3	Other
Dok3	Docking Protein 3	Immune system
Ecm1	Extracellular Matrix Protein 1	Cell adhesion
Efemp1	EGF Containing Fibulin Extracellular Matrix Protein 1	Cell adhesion
Gbe1	1,4-Alpha-Glucan Branching Enzyme 1	Proteoglykan synthesis
Gngt2	G Protein Subunit Gamma Transducin 2	Other
Golga4	Golgin A4	Development of nervous system
Gria1	Glutamate Ionotropic Receptor AMPA Type Subunit 1	Neuronal signaling
Grik4	Glutamate Ionotropic Receptor Kainate Type Subunit 4	Neuronal signaling
Hkdc1	Hexokinase Domain Containing 1	Glycogen synthesis
Hmox1	Heme Oxygenase 1	Other
Hrc	Histidine Rich Calcium Binding Protein	Other
Htr1b	5-Hydroxytryptamine Receptor 1B	Neuronal signaling
Icam5	Intercellular Adhesion Molecule 5	Immune system
Il12rb1	Interleukin 12 Receptor Subunit Beta 1	Immune system
Il23a	Interleukin 23 Subunit Alpha	Immune system
Il33	Interleukin 33	Immune system
Inpp5j	Inositol Polyphosphate-5-Phosphatase J	Development of nervous system
Kcnip1	Potassium Voltage-Gated Channel Interacting Protein 1	Ion transport
Lbp	Lipopolysaccharide Binding Protein	Immune system
Lcat	Lecithin-Cholesterol Acyltransferase	Other
Lcn2	Lipocalin 2	Immune system
Lrp4	LDL Receptor Related Protein 4	Neuronal signaling
Ltbp2	Latent Transforming Growth Factor Beta Binding Protein 2	Cell adhesion
Matn3	Matrilin 3	Cell adhesion
Met	MET Proto-Oncogene, Receptor Tyrosine Kinase	Cellular growth
Mmp19	Matrix Metallopeptidase 19	Cell adhesion
Mt1	Metallothionein 1	Stress response
Myl4	Myosin Light Chain 4	Other
Ndn	Necdin, MAGE Family Member	Development of nervous system
Nedd4l	NEDD4 Like E3 Ubiquitin Protein Ligase	Ubiquitine
Nod2	Nucleotide Binding Oligomerization Domain Containing 2	Immune system
Npas2	Neuronal PAS Domain Protein 2	Other
Nt5e	5’-Nucleotidase Ecto	Immune system
Ntn1	Netrin 1	Differentiation
Osmr	Oncostatin M Receptor	Cell division
P2rx1	Purinergic Receptor P2X 1	Neuronal signaling
P2rx6	Purinergic Receptor P2X 6	Neuronal signaling
Padi2	Peptidyl Arginine Deiminase 2	Cell differentiation
Pak1	P21 (RAC1) Activated Kinase 1	Cell differentiation
Pdgfb	Platelet Derived Growth Factor Subunit B	Cellular growth
Plch2	Phospholipase C Eta 2	Neuronal signaling
Pou3f1	POU Class 3 Homeobox 1	Cell differentiation
Pou3f2	POU Class 3 Homeobox 2	Cell differentiation
Ptpn6	Protein Tyrosine Phosphatase Non-Receptor Type 6	Cell differentiation
Qpct	Glutaminyl-Peptide Cyclotransferase	Neuronal signaling
Rab18	RAB18, Member RAS Oncogene Family	Neuronal development
Rap2c	RAP2C, Member Of RAS Oncogene Family	Cell differentiation
Rarres2	Retinoic Acid Receptor Responder 2	Immune system
Rgs6	Regulator Of G Protein Signaling 6	Other
Rims1	Regulating Synaptic Membrane Exocytosis 1	Vesicular exocytosis
Ripk3	Receptor Interacting Serine/Threonine Kinase 3	Apoptosis
Scn1b	Sodium Voltage-Gated Channel Beta Subunit 1	Neuronal signaling
Scube3	Signal Peptide, CUB Domain And EGF Like Domain Containing 3	Development of nervous system
Sema3e	Semaphorin 3E	Neuronal development
Slc17a7	Solute Carrier Family 17 Member 7	Neuronal signaling
Slc22a3	Solute Carrier Family 22 Member 3	Neuronal signaling
Slc7a11	Solute Carrier Family 7 Member 11	Metabolism
Slit1	Slit Guidance Ligand 1	Neuronal development
Sncb	Synuclein Beta	Development of nervous system
Sparcl1	SPARC Like 1	Neuronal development
Stat6	Signal Transducer And Activator Of Transcription 6	Immune system
Thrsp	Thyroid Hormone Responsive	Metabolism
Tnni1	Troponin I1, Slow Skeletal Type	Other
Trpm2	Transient Receptor Potential Cation Channel Subfamily M Member 2	Neuronal signaling
Trpm7	Transient Receptor Potential Cation Channel Subfamily M Member 7	Neuronal signaling
Trpv4	Transient Receptor Potential Cation Channel Subfamily V Member 4	Neuronal signaling
Txnip	Thioredoxin Interacting Protein	Glycogen synthesis
Vps37a	VPS37A Subunit Of ESCRT-I	Ubiquitine
Vps41	VPS41 Subunit Of HOPS Complex	Vesicular exocytosis

**Table 4:** Genes of interest from MASER14, down-regulated in cells exposed to space flight.

Gene symbol	Gene description	Assigned function
Alox5	Arachidonate 5-Lipoxygenase	Immune system
Bsn	Bassoon Presynaptic Cytomatrix Protein	Vesicle biosynthesis
Casp1	Caspase 1	Immune system
Cftr	*CF* Transmembrane Conductance Regulator	Other
Cnih3	Cornichon Family AMPA Receptor Auxiliary Protein 3	Neuronal signaling
Cntn6	Contactin 6	Cell adhesion
D2hgdh	D-2-Hydroxyglutarate Dehydrogenase	Mitochondria
Fzd6	Frizzled Class Receptor 6	Cell division
Gdap1	Ganglioside Induced Differentiation Associated Protein 1	Mitochondria
Gja10	Gap Junction Protein Alpha 10	Cell–Cell adhesion
Gja4	Gap Junction Protein Alpha 4	Cell–Cell adhesion
Grb10	Growth Factor Receptor Bound Protein 10	Cell division
Grik1	Glutamate Ionotropic Receptor Kainate Type Subunit 1	Neuronal signaling
Hif3a	Hypoxia Inducible Factor 3 Subunit Alpha	Hypoxia
Igf2	Insulin Like Growth Factor 2	Cell division
L3mbtl1	L3MBTL Histone Methyl-Lysine Binding Protein 1	Cell division
Loxl4	Lysyl Oxidase Like 4	Collagen synthesis
Lpar3	Lysophosphatidic Acid Receptor 3	Differentiation
Mag	Myelin Associated Glycoprotein	Cell–Cell adhesion
Mboat4	Membrane Bound O-Acyltransferase Domain Containing 4	Other
Myh11	Myosin Heavy Chain 11	Other
Mylk	Myosin Light Chain Kinase	Other
Myt1	Myelin Transcription Factor 1	Development of nervous system
Nkx2-2	NK2 Homeobox 2	Development of nervous system
Osr1	Odd-Skipped Related Transcription Factor 1	Development of nervous system
Pgr	Progesterone Receptor	Other
Pla2g4a	Phospholipase A2 Group IVA	Immune system
Rab33a	RAB33A, Member RAS Oncogene Family	Vesicle biosynthesis
Rhd	Rh Blood Group D Antigen	Other
Rspo3	R-Spondin 3	Cell division
Sftpc	Surfactant Protein C	Other
Slc17a8	Solute Carrier Family 17 Member 8	Neuronal signaling
Slc30a10	Solute Carrier Family 30 Member 10	Other
Slc7a8	Solute Carrier Family 7 Member 8	Metabolism
Slc9a2	Solute Carrier Family 9 Member A2	Other
Spn	Sialophorin	Immune system
Sptbn2	Spectrin Beta, Non-Erythrocytic 2	Cell division
Stx3	Syntaxin 3	Neuronal signaling
Trpm3	Transient Receptor Potential Cation Channel Subfamily M Member 3	Neuronal signaling
Vtn	Vitronectin	Cell adhesion
Wnt2b	Wnt Family Member 2B	Development of nervous system
Zp1	Zona Pellucida Glycoprotein 1	Other

The genes of interest were assigned a categorical classification based on functional classification in gene ontology using the DAVID tool (Huang da et al., 2009a,b) and the NCBI gene info database.^3^ Further, the predicted cellular localization was obtained using the WegoLoc tool (Chi and Nam, 2012). Data were visualized using MS Excel and Graph Pad Prism V5.0 (GraphPad Software Inc., Boston, MA, United States).

### 2.5 Exosome analysis

#### 2.5.1 Isolation of BC-derived exosomes

BCs were maintained in culture in DMEM/F12 medium supplemented with N2 and B27 (see above). After the specimens were delivered to the Esrange laboratory, the medium was collected for exosome analysis. Exosome isolation was performed using Amicon^®^ Ultra-15 Centrifugal Filter Unit with Ultracel-100 regenerated cellulose membrane (UFC910024, Millipore, Massachusetts, United States). The cellular medium was centrifuged at 2000 rcf for 30 min at 4°C and washed with PBS at 2000 rcf for 30 min at 4°C. Exosomes kept by the filter were then collected and stored at −20°C.

#### 2.5.2 Exosome observations—transmission electron microscopy

Exosome fixation for TEM was performed using 2% glutaraldehyde solution in phosphate buffer (ratio 1:1). After the fixation phase, exosomes were deposited, rinsed, and stained with heavy metal compounds onto a gridded slide following standard protocols. The visualization of the slide was performed using a TEM Zeiss EM 910 instrument (Zeiss, Oberkochen, Germany).

#### 2.5.3 Exosome characterization with tunable resistive pulse sensing

BC exosome distribution and diameter size were analyzed with the qNano platform (iZON Science, UK). NP150 nanopores and CPC200 calibration particles were used to analyze at 20 mbar pressure. The output was then analyzed with the Izon control suite v3.4 software, which allows for comparing the sample and calibration particles as a comparative reference.

#### 2.5.4 Total exosome RNA extraction and miRNA sequencing

Total RNA extraction from the BC-derived exosomes was performed using the Cell Culture Media Exosome Purification and RNA Isolation Mini Kit (Norgen Biotek Corp., Thorold, Ontario, Canada), following the manufacturer’s instructions. All RNA samples were then stored at −80°C.

Illumina sequencing was used to realize miRNA profiling, which was carried out by Area Science Park (ASP, Trieste, Italy). MiRNA-Seq libraries were realized using the QIAseq miRNA Library Kit (QIAGEN; Hilden, Germany). The sequencing was performed using Novaseq 6000 (Illumina; San Diego, CA, United States) in the 2 × 150 paired-end mode. The identification of miRNAs in the samples was done using the QIAseq miRNA-NGS data analysis software V5, considering single read as the read type and Read 1 Cycles 75 as the read cycles.

#### 2.5.5 Bioinformatic and statistical analysis

MiRNAs from QIAseq miRNA-NGS data analysis software were selected based on read number. The final list of miRNAs obtained was used in enrichment analysis using miRNet software (Chang and Xia, 2023). Functional enrichment analysis of miRNA was realized using miRTareBase v8.0 database as reference. The software was exploited to perform a Gene Ontology Biological Process Enrichment. A *p*-value < 0.05 was chosen to select data, and Prism 8.03 software graphical view (GraphPad Software Inc., Boston, MA, United States) was used to report enrichment analysis.

**Figure 2:**
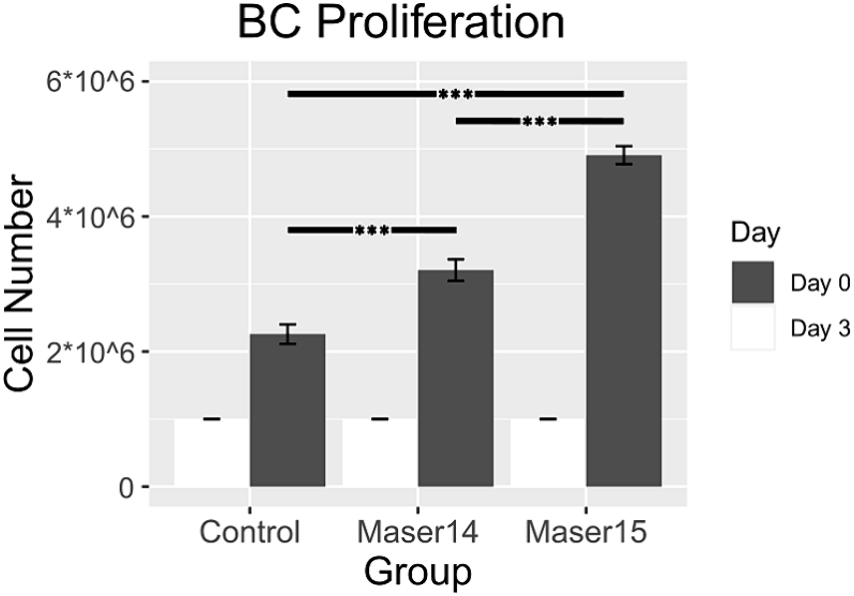
BC proliferation between the ground control group, the cells that were aboard the MASER14, and BC aboard the MASER15 on the seeding day (Day 0) and Day 3 of culture (*p < 0.05, **p < 0.01, ***p < 0.001, as per One-Way ANOVA and Tukey’s HSD *post-hoc* test).

For evaluation of BC proliferation, statistics such as One-Way ANOVA and Tukey’s HSD *post-hoc* test were calculated in RStudio Version 4.0.5. Additionally, the package “ggplot2” was used to plot the graph (Figure 2).

## 3 Results

### 3.1 Space flown BCs show enhanced proliferation

We previously reported that BCs increased their proliferation capacity after a space flight with the sounding rocket MASER14 (Han et al., 2021), for which experimental material was harvested 1 week after landing (delayed harvest). All material from the MASER15 experiment was collected directly after the flight (immediate harvest). Control BCs and BCs from MASER14 and MASER15 μg groups were split into single cells, seeded at 0.3 M cells/mL, and cultured for 3 days, and the number of cells was assessed at the end of the experiment. The results show that 5 h after flight harvested BCs proliferated significantly faster than BCs harvested after a 1 week delay, as well as control BCs (Figure 2).

### 3.2 Space flown BCs show altered gene expression

Whole transcriptome profiles obtained using the AmpliSeq method for BCs exposed to μg on MASER14 and MASER15 were compared to their corresponding ground control groups. For MASER15, 104 genes were differentially expressed (log2FC > 2.0, FDR adjusted *p* < 0.001) in μg compared to ground control (42 upregulated and 62 downregulated genes). For MASER14, 479 genes met the fold change limit (334 upregulated and 145 downregulated). BCs from MASER14 were harvested 7 days after μg, while BCs from MASER15 were harvested directly after the flight (5–6 h after μg exposure). Hence, potential differences in the effect on gene expression in relation to time after μg exposure could be evaluated.

**Figure 3:**
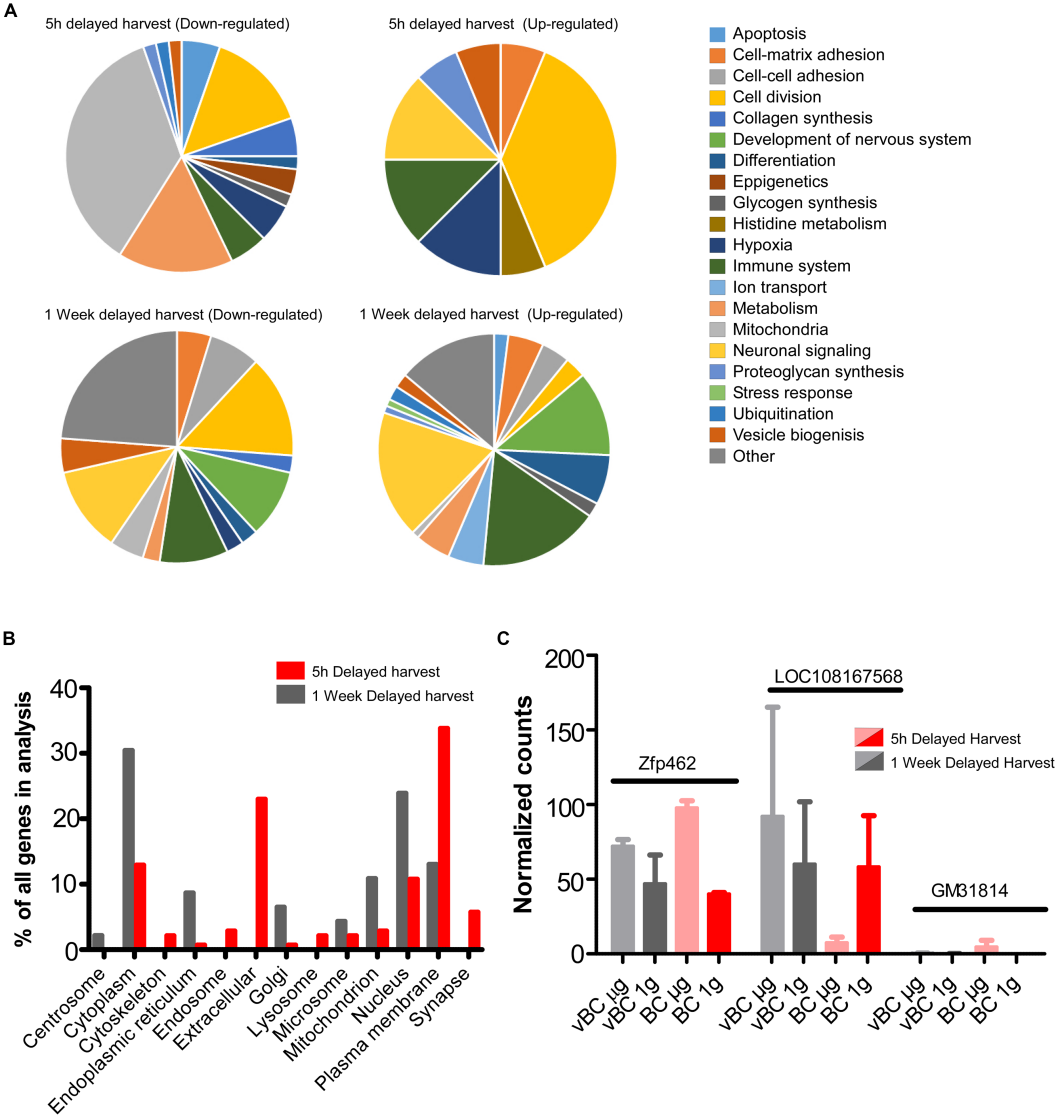
**(A)** Classification of significantly enriched genes according to Reactome pathways. **(B)** Classification of significantly changed genes into predicted cellular localization according to WegoLoc classification. **(C)** Genes with significantly changed expression between 5 h harvest at 1G and 5 h harvest at μg.

The differentially expressed genes were assessed using the REACTOME pathway database (Fabregat et al., 2017). In MASER15 samples, pathways related to disease, gene expression, signal transduction, cell cycle, and programmed death were found to be enriched (Tables 1, 2). At the same time, in MASER14, enrichment of pathways related to the immune system, transport of small molecules, cellular response to stimuli, and metabolism of proteins were detected (Tables 3, 4). Whether these differences in gene expressions are due to the delayed effect of μg in MASER14 or the changes in stem cells due to the prolonged effect of μg may be addressed in future space experiments with BCs. Genes defining the enrichment of the listed pathways were extracted and used for further analysis (Tables 1–4). The genes were further grouped into 21 classes, including one class named “Other” for singular genes with more deviant categorization, based on the REACTOME enrichment analysis (Figure 3A).

Following immediate harvest, the largest groups of upregulated genes were related to proliferation, hypoxia, and immune signaling. Further, genes related to metabolism and proliferation were also most downregulated, emphasizing the impact of space flight on proliferation. Interestingly, we also detected downregulation of genes that prevent epigenetic changes. After delayed harvest, upregulation of genes involved in stress response and development of the nervous system and downregulation of genes involved in cell division and development of the nervous system were detected.

When genes were classified according to cellular localization, there was a higher number of genes localized to the cytoplasm and nucleus in immediately harvested BCs, while the number of genes localized to the plasma membrane and synapses was increased in delayed harvested BCs, compared to ground controls (Figure 3B). Interestingly, a relatively large number of genes on the MASER14 gene lists were found to be extracellular, e.g., neuropeptides, growth factors and cytokines (Table 3).

The MASER15 μg group was further compared to the MASER15 1 g control, which was subjected to all aspects of the flight except μg (Figure 2). Here, only five differentially expressed genes were found (FDR < 0.05, Log2FC > 1.0): 9530082P21Rik, Uox, Zfp462, Malat1, Gm31814, and LOC108167568. Three genes (9530082P21Rik, Uox, and Gm31814) were excluded from further assessment due to expression in only one sample. Upregulated genes were Zfp462, that encodes a zink-finger protein known to regulate survival in early development, and Malat1, which produces a precursor to a non-coding RNA. LOC108167568, encoding a transcription factor without known function but with an active binding site, was found to be downregulated (Figure 3C).

### 3.3 BC exosome number and morphology differ after direct compared to delayed harvest

**Figure 4:**
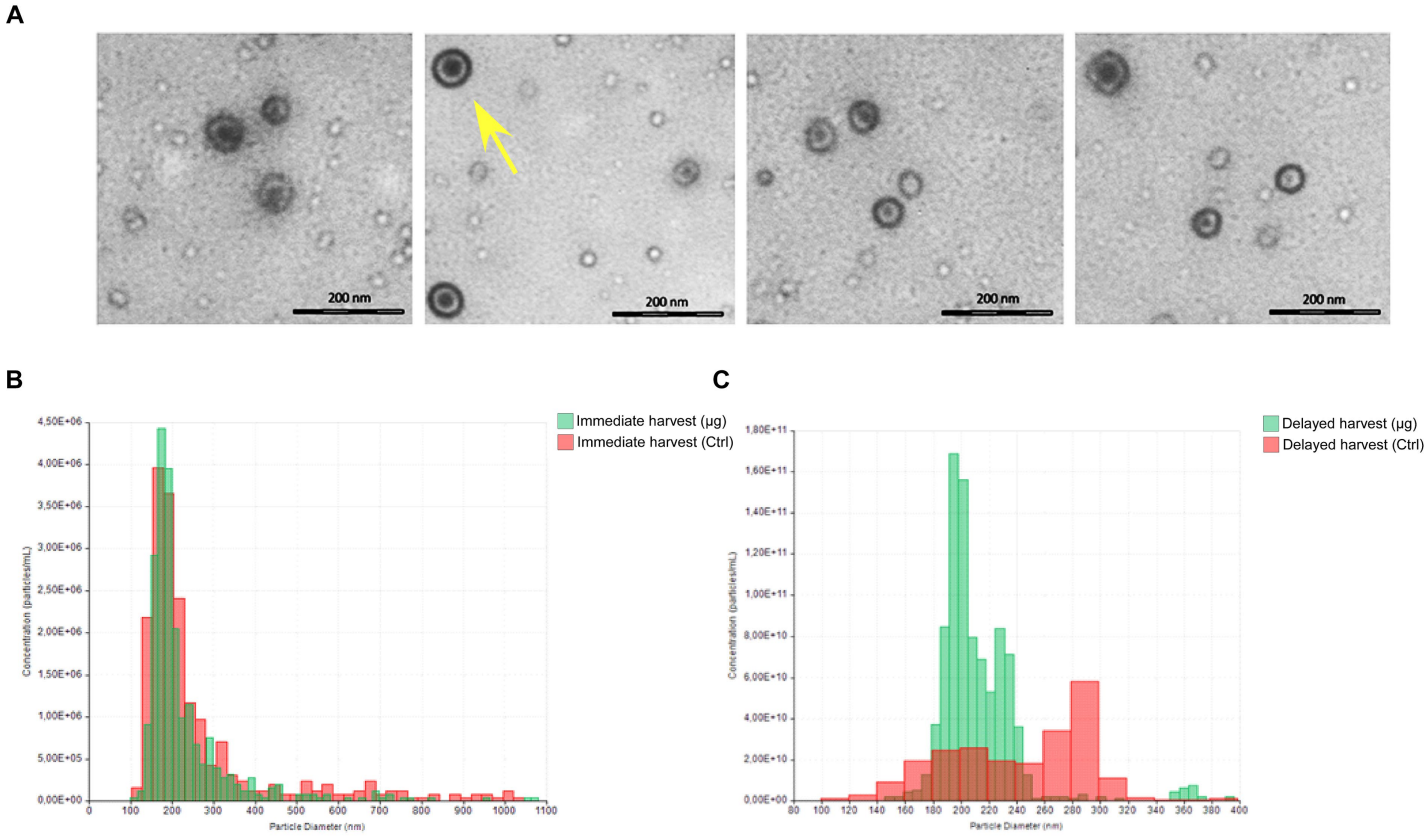
Total BC-derived exosomes characterization. **(A)** Representative TEM image for each samples. **(B,C)** Size distribution and concentration using tunable resistive pulse sensing instrument qNano (iZON Science, Oxford, UK).

Exosomes were isolated from BC μg and BC ground control medium and characterized. First, they were observed using transmission electron microscopy (TEM). The resulting extracellular vesicles showed a typical bilayer cup-shaped membrane structure, appearing like rounded structures in TEM (Figure 4A). Tunable resistive pulse sensing analysis was used to measure the dimension of the vesicles (Table 5; Figures 4B,C). The analysis of exosome sizes shows similarity between MASER14 and MASER15 μg groups, similar to the control group of MASER15 (directly collected medium after μg exposure). In the MASER14 group, when the control medium was collected with a delay of 1 week, we detected fewer, but larger exosomes (Figure 4C).

**Table 5:** Average exosome size and concentration in MASER14 and MASER15 BC µG samples, and in corresponding BC control samples.

Sample	Diameter average (nm)	Concentration
BC μg (MASER15)	231	1.07e+07
BC Ctrl (MASER15)	265	9.38e+06
BC μg (MASER14)	212	9.17e+11
BC Ctrl (MASER14)	241	2.26e+11

### 3.4 Exosomes from space flown BCs differ from controls in their miRNA content

**Figure 5:**
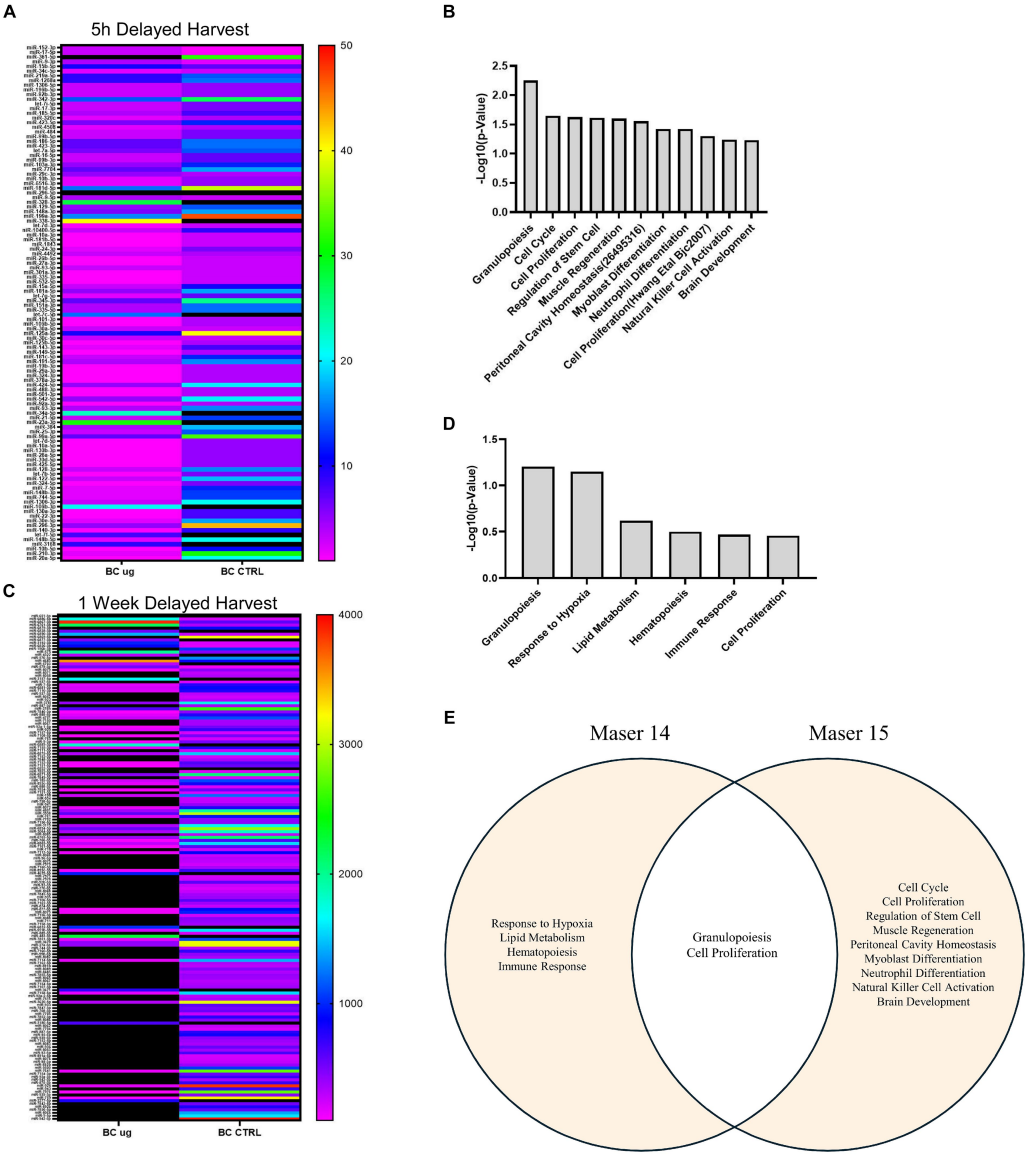
miRNAs expression profiling analysis. **(A)** HeatMap on the reads of 110 significant miRNAs of BC μg vs. BC Ctrl (immediate harvest) comparison. **(B)** MiRNet biological function enrichment on up-regulated of BC μg vs. BC Ctrl (immediate harvest) miRNAs [significant value reported as −Log10(*p*-value)]. **(C)** HeatMap on the reads of significant miRNAs of BC μg vs. BC 1G on board (immediate harvest) comparison. **(D)** MiRNet biological function enrichment on up-regulated of BC μg vs. BC 1G on board (immediate harvest) miRNAs [significant value reported as −Log10(*p*-value)]. **(E)** Venn diagram describing the common pathways between Maser14 and Maser15 experiments.

After the isolation, all the exosome content was extracted to perform miRNA sequencing analysis. From the miRNA sequencing analysis of the immediately harvested MASER15 BC μg samples compared to corresponding ground control, 110 miRNAs showed a significant fold-regulation value (cut off: < −2 or > +2) (Figure 5A). Among these significantly altered miRNAs, five were upregulated: miR-152-3p, miR-17-5p, miR-15b-5p miR-361-5p, and miR-9-3p. Of these miRNAs, the first three are involved as regulators of biological processes including proliferation, extracellular matrix production, and apoptosis (Cloonan et al., 2008; Gan et al., 2021; Pinazo-Duran et al., 2023), while miR-361-5p and miR15b-5p are reported to be tumor suppressors and brain-specific miRNA (Ji et al., 2016; Ma et al., 2017; Zhou et al., 2022).

To further explore the function of upregulated, an enrichment analysis was performed using miRNet software. From the miRNet output, only the biological processes with a *p*-value < 0.05 were selected and reported in a bar graph (Figure 5B). The analysis performed on up-regulated miRNAs shows enrichment in immuno-modulating functions, cell cycle/proliferation mechanism, regulation of stem cells, and brain development. The miRNA sequencing analysis was also performed for MASER15 μg sample compared with the 1 g on board control sample. This analysis yielded 48 significantly altered miRNAs (cut-off: < −1.5 or > +1.5). Of these, 12 were down-regulated and 36 up-regulated in MASER15 μg sample compared to 1 g sample. Among the up-regulated miRNAs is reported let-7b-5p, that represents a regulator of Zfp462 gene resulted as a significant gene in the gene expression analysis.

An identical miRNA analysis performed on BC μg and related control samples following delayed harvest from MASER14, showed a total of 169 significantly altered miRNAs based on fold-regulation value (cut off: < −2 or > +2) (Figure 5C). Eighteen of these miRNAs (miR-651-3p, miR-6886-5p, miR-6867-3p, miR-6761-3p, miR-6879-3p, miR-6828-3p, miR-6890-3p, miR-6869-5p, miR-6877-3p, miR-3192-3p, miR-6826-3p, miR-196b-3p, miR-575, miR-4322, miR-570-3p, miR-4683, miR-5703, miR-579-3p) were upregulated in BC μg condition compared to control. An enrichment analysis of these 18 upregulated miRNAs revealed their involvement granulopoiesis, response to hypoxia, lipid metabolism, hematopoiesis, immune response, and cell proliferation (Figure 5D). A visual Venn Diagram was exploited to highlight the common biological pathways between MASER 14 and MASER 15 (Figure 5E).

**Figure 6:**
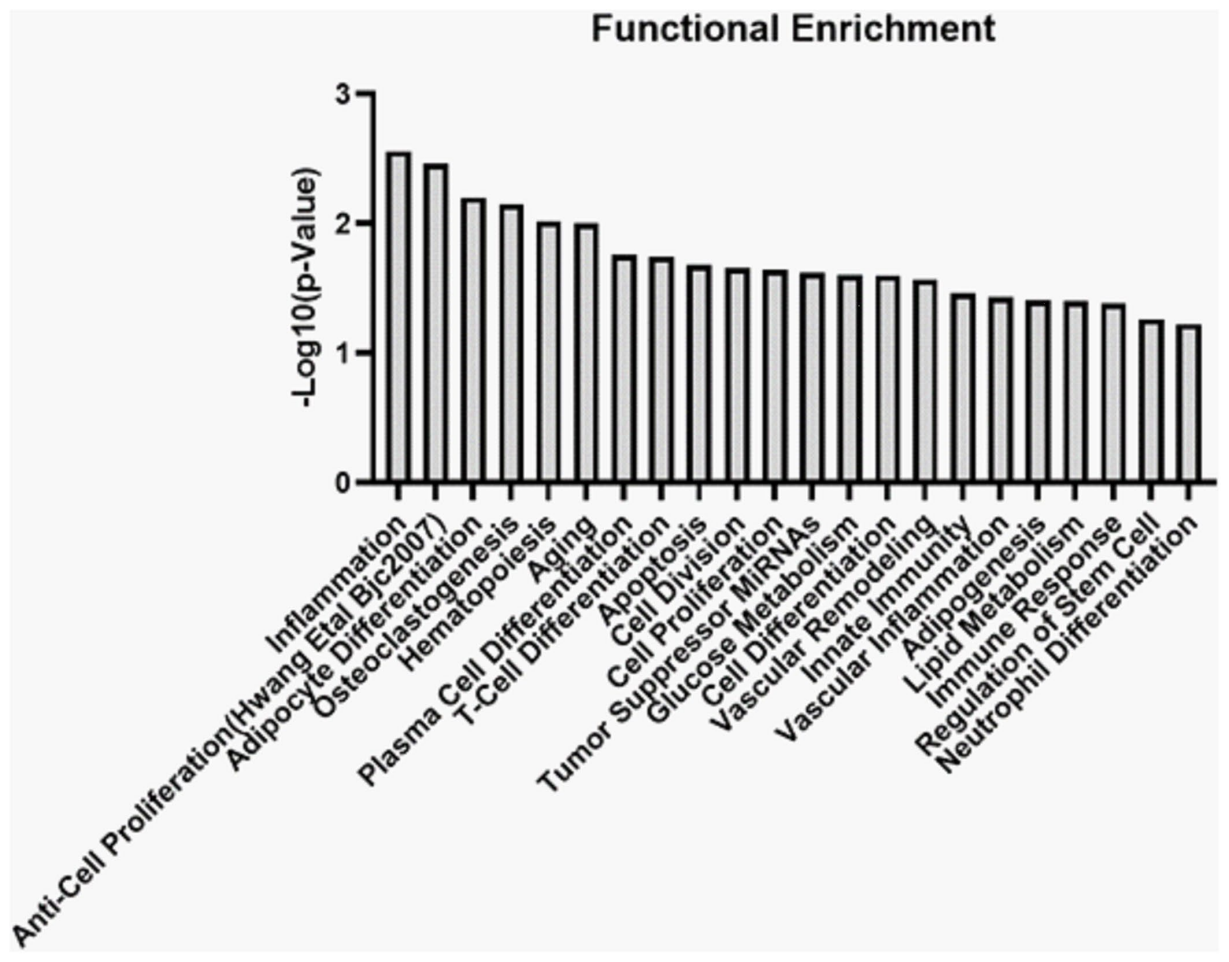
MiRNet biological function enrichment on 46 common miRNAs [significant value reported as −Log10(*p*-value)].

Furthermore, overlap analysis performed on the list of miRNAs from MASER14 and MASER15 BC μg condition resulted in the identification of 46 common miRNAs in the samples analyzed (Table 6). An enrichment analysis on these common miRNAs to verify a possible biological process enrichment pattern shows their involvement in immune system mechanisms, differentiation, proliferation and regenerative processes, as well as in glucose and lipid metabolisms (Figure 6).

**Table 6:** Common miRNAs in MASER14 and MASER15 BC µg samples.

Common miRNAs	
let-7b-5p	miR-20a-5p
let-7c-5p	miR-23a-3p
let-7d-5p	miR-24-3p
let-7f-5p	miR-25-3p
let-7 g-5p	miR-26a-5p
let-7i-5p	miR-296-3p
miR-101-3p	miR-29a-3p
miR-103a-3p	miR-301a-3p
miR-10a-5p	miR-335-5p
miR-125a-5p	miR-342-3p
miR-125b-5p	miR-34a-5p
miR-1268a	miR-361-5p
miR-128-3p	miR-378a-3p
miR-130a-3p	miR-423-3p
miR-148a-3p	miR-424-5p
miR-148b-3p	miR-532-5p
miR-151a-3p	miR-744-5p
miR-152-3p	miR-92a-3p
miR-16-5p	miR-93-5p
miR-181a-5p	miR-9-3p
miR-181b-5p	miR-9-5p
miR-191-5p	miR-99a-5p
miR-196b-5p	miR-99b-5p

## 4 Discussion

We show that a short flight with sounding rocket enhances the proliferation capacity by BCs and alters their gene expression and exosome miRNA content. The different patterns of gene expression in samples collected 5 h or 1 week after the exposure to μg compared to ground control groups, show that some post-microgravity effects can be detected directly after the flight, whereas other effects can appear later.

We previously showed a markedly increased proliferation capacity in BCs flown with MASER14 sounding rocket (Han et al., 2021). MASER15 BCs showed a remarkably high proliferation rate compared to 1 g samples on board, indicating that this feature was a result specifically of μg exposure during sounding rocket flight. Genetic analysis of the same two groups of MASER15 BCs revealed significant changes in three genes. The Zfp462, which encodes a zink-finger protein known to regulate survival in early development and contribute to cell proliferation (Yelagandula et al., 2023), was up-regulated in μg exposed BCs. Zfp462 secures neural lineage specification of mouse embryonic stem cells (ESCs) by silencing mesoendodermal genes due to targeting histone methyltransferase complex and direct epigenetic regulation.

The other two genes, Malat1 and LOC108167568, are encoding a precursor to a non-coding RNA and a transcription factor without known function, respectively. The change in expression of these genes could initiate increased proliferation and survival in μg-exposed cells at later stages after μg. The role of non-coding RNAs is now under extensive investigation by several groups, and their potential role in gene activation and protein synthesis is suggested (Kehl et al., 2017; Balashanmμgam et al., 2019; Hwang et al., 2023). It has been shown that overexpression of Zfp462 is essential for the development of the nervous system (Chang et al., 2007; Laurent et al., 2009). Zfp462 has also been shown to be crucial for maintaining stemness (Masse et al., 2010, 2011; Yelagandula et al., 2023).

The exosome-derived miRNAs profile from the MASER15 experiment showed up-regulation of the miRNA let-7b-5p in the μg sample compared to 1 g according to the miRTareBase, a reference database for target genes of miRNAs, Zfp462it is one of the target genes of let-7b-5p (Huang et al., 2020). The up-regulation of this miRNA might be expected to decrease or block the expression of Zfp462. However, given the complex effects of μg exposure, with alterations in the activity of multiple genes and changes in miRNA exosomal content, the precise influence of this miRNA is speculative.

When gene activity was compared between MASER14 and MASER15, it was found that protein activity locations are distributed differently. After the flight in MASER15 cells, the primary localization was detected mostly inside the nuclei and the cytosol. In contrast, after the MASER14 flight, the activity of the proteins was localized in the cell membrane and associated with the secretion. This distribution suggests that cells concentrate their activity on their survival during flight. Still, after the flight, they possess new features related to cell–cell communication, which may reflect their supporting characteristics in co-culture (Grouwels et al., 2012; Ngamjariyawat et al., 2013; Aggarwal et al., 2017) and co-transplantation with other cells (Olerud et al., 2009; Grapensparr et al., 2015; Aggarwal et al., 2017).

The differences in gene expression between MASER15 and MASER14 showed that early gene activity in MASER15 was associated with cell division and downregulation of cell adhesion, whereas in MASER14 a gene activity related to pro-inflammatory response and, to a lesser extent, cell division was enriched. In MASER14, an upregulation of genes related to the immune system was detected. However, this designation was a result of Toll-Like Receptor (TLR) activation, which may be associated with cell defense mechanisms in non-immune cells (Song et al., 2019). The extreme resistance of BCs to external stress factors may partly reflect the upregulation of these genes.

The analysis of the number and size of exosomes from MASER14 and MASER15 medium revealed that compared to control cells cultured on the ground, the size in the MASER15 group did not differ. In contrast, in the control group of MASER14, the size of exosomes increased, whereas the number of exosomes was reduced. These findings indicate that the size of exosomes exposed to μg did not change and remained similar to the ground control exosomes, as shown after direct harvest. In contrast, exosomes from MASER14 medium, when cells were in post-flight condition for 1 week, underwent significant modification, suggesting that μg supports maintenance of baseline exosome production, similar to ground control.

We also analyzed miRNA content in the exosomes from the medium of the MASER14 and MASER15 groups. MiRNAs identified in MASER14 exosomes are involved in processes like proliferation, cell cycle, and regulation of stem cell fate. In contrast miRNA exosomes from MASER15 were found to be associated with protection from hypoxia and, to a lesser extent, with cell proliferation (Yan et al., 2020; Wei et al., 2022). These results correlate with the significantly increased proliferation of cells after space flight, as well as the pathway enrichment analysis of the transcriptomic data showing an altered gene expression related to cell proliferation. The mechanisms underlying possible delayed emergence of space flight-induced changes in cellular properties are most likely due to altered gene regulation, e.g., DNA methylation or histone modifications. These alterations may, in turn, lead to a long-lasting change in cell properties, either as a lowered cell intrinsic threshold for entering the cell cycle or by inducing the release of factors that operate in an autocrine or paracrine manner to stimulate proliferation.

There are numerous reports, based on different types of actual and simulated μg exposure, of long-term up- and downregulation of genes and of alterations in the expression of regulatory molecules such as miRNAs in a range of cell types (Corydon et al., 2023). These alterations reflect adaptations associated with cellular stress, but a correlation with beneficial effects has also been demonstrated. Our previous study on BCs exposed to sounding rocket MASER14 flight showed an upregulation of genes related to proliferation and survival (Han et al., 2021). Remarkably, MASER14-flown BCs still showed an increased proliferation rate compared to control BCs 3 years after space flight, though not as high as MASER15 BCs. This agrees with the exosome analysis of MASER14, where miRNAs related to proliferation were altered after delayed harvest, indicating a lingering effect on cell growth.

We conclude that neural crest stem cells increase their proliferation capacity after space flight due to exposure to μg, an outcome that can be detected immediately after space flight, as well as in specimens harvested after a delay. This effect is associated with alterations in gene expression, among which upregulation of the transcription factor Zfp462 may be particularly relevant for the observed increased proliferation capacity. We find a complex pattern of regulation of additional genes, as well as exosomal miRNAs, including regulators involved in cell stress response. Further studies in ground-based simulated and prolonged space μg experiments will help to elucidate the mechanisms of direct and delayed effects of μg and elucidate the metabolic characteristics of BCs during flight conditions, which underlie their remarkable survival capacity in stress conditions. This will improve our understanding of the impact of μg on neural stem cells or other type of cells, and contribute to potential clinical application, such as approaches for controlled and rapid cell renewal for cell replacement therapy and tissue engineering.

## Data availability statement

The raw data supporting the conclusions of this article will be made available by the authors, without undue reservation.

## Ethics statement

The animal study was approved by Uppsala Regional Committee for the Care and Use of Animals in Research. The study was conducted in accordance with the local legislation and institutional requirements.

## Author contributions

YH: Data curation, Formal analysis, Investigation, Methodology, Validation, Visualization, Writing – review & editing. PB: Data curation, Formal analysis, Investigation, Methodology, Validation, Visualization, Writing – review & editing. LZ: Data curation, Formal analysis, Investigation, Validation, Writing – review & editing. SS: Data curation, Formal analysis, Funding acquisition, Investigation, Methodology, Software, Validation, Visualization, Writing – review & editing. FZ: Data curation, Formal analysis, Funding acquisition, Investigation, Methodology, Validation, Visualization, Writing – review & editing. ME: Conceptualization, Formal analysis, Resources, Validation, Writing – review & editing. BZ: Data curation, Formal analysis, Investigation, Resources, Supervision, Validation, Visualization, Writing – review & editing. MT: Data curation, Formal analysis, Investigation, Methodology, Writing – review & editing. GF: Data curation, Formal analysis, Validation, Visualization, Writing – review & editing. AV: Investigation, Software, Validation, Writing – review & editing. HA: Data curation, Formal analysis, Project administration, Validation, Writing – review & editing. RF: Conceptualization, Investigation, Methodology, Software, Visualization, Writing – original draft. EK: Conceptualization, Funding acquisition, Methodology, Resources, Supervision, Visualization, Writing – original draft.

## Footnotes

^1^ https://www.sscspace.com

^2^ https://www.siouxtechnologies.com

^3^ https://www.ncbi.nlm.nih.gov/gene
